# Supplementary material for: Deletion of wfs1 Impairs Oligodendrocyte Precursor Cells Dorsal Distribution and Myelination Through the wfs1-hmgcs1 Axis in Zebrafish
Source: Biology (Basel). 2026 Aug 16;15(16):1402. doi: 10.3390/biology15161402 (PMC13510151; doi:10.3390/biology15161402)
Supplement: Supplementary file 1 [file biology-15-01402-s001.zip › File S1-Transcriptome sequencing data and differentially expressed genes (DEGs) in wfs1-- zebrafish at 5 dpf/1.GO/wfs1KOvsWT/GSEA/WT/AUTOPHAGOSOME_MATURATION/AUTOPHAGOSOME_MATURATION(GO_0097352).html]

Details for gene set AUTOPHAGOSOME\_MATURATION(GO:0097352)[GSEA]


|  || Dataset | deg.sample |
| Phenotype | sample.cls |
| Upregulated in class | WT |
| GeneSet | AUTOPHAGOSOME\_MATURATION(GO:0097352) |
| Enrichment Score (ES) | -0.54951364 |
| Normalized Enrichment Score (NES) | -1.8249984 |
| Nominal p-value | 0.0 |
| FDR q-value | 0.23552263 |
| FWER p-Value | 0.607 |
Table: GSEA Results Summary

  

Fig 1: Enrichment plot: AUTOPHAGOSOME\_MATURATION(GO:0097352)
  
Profile of the Running ES Score & Positions of GeneSet Members on the Rank Ordered List

  

| PROBE | DESCRIPTION   (from dataset) | GENE SYMBOL | GENE\_TITLE | RANK IN GENE LIST | RANK METRIC SCORE | RUNNING ES | CORE ENRICHMENT || 1 | 492808 | stx17 |  |  | 3434 | 0.945 | -0.0397 | No |
| 2 | 100330105 | map1lc3cl |  |  | 5232 | 0.652 | -0.0492 | No |
| 3 | 541406 | lamp2 |  |  | 7906 | 0.431 | -0.1020 | No |
| 4 | 406466 | map1lc3a |  |  | 9731 | 0.319 | -0.1362 | No |
| 5 | 558010 | lix1 |  |  | 12304 | 0.174 | -0.2045 | No |
| 6 | 555970 | snx14 |  |  | 13277 | 0.126 | -0.2258 | No |
| 7 | 100004597 | phf23b |  |  | 13280 | 0.126 | -0.2168 | No |
| 8 | 100329883 | fyco1b |  |  | 13392 | 0.121 | -0.2116 | No |
| 9 | 556500 | tecpr1a |  |  | 16379 | -0.013 | -0.3043 | No |
| 10 | 556338 | tbc1d25 |  |  | 18255 | -0.088 | -0.3568 | No |
| 11 | 324307 | atp6v0a1a |  |  | 18391 | -0.093 | -0.3544 | No |
| 12 | 393184 | clec16a |  |  | 18413 | -0.094 | -0.3482 | No |
| 13 | 565649 | fyco1a |  |  | 18448 | -0.096 | -0.3424 | No |
| 14 | 563679 | zgc:136908 |  |  | 18750 | -0.111 | -0.3438 | No |
| 15 | 100000896 | rubcn |  |  | 18805 | -0.114 | -0.3373 | No |
| 16 | 561574 | si:ch211-260b17.6 |  |  | 19278 | -0.134 | -0.3425 | No |
| 17 | 100151338 | tecpr1b |  |  | 19485 | -0.143 | -0.3386 | No |
| 18 | 407723 | smcr8b |  |  | 19750 | -0.155 | -0.3357 | No |
| 19 | 323208 | tmem39a |  |  | 20698 | -0.199 | -0.3512 | No |
| 20 | 393268 | map1lc3c |  |  | 27020 | -0.631 | -0.5041 | Yes |
| 21 | 322425 | map1lc3b |  |  | 28058 | -0.750 | -0.4826 | Yes |
| 22 | 572161 | lix1l |  |  | 28784 | -0.875 | -0.4423 | Yes |
| 23 | 799873 | spns1 |  |  | 31576 | -3.178 | -0.3010 | Yes |
| 24 | 327197 | vcp |  |  | 31772 | -4.315 | 0.0037 | Yes |
Table: GSEA details
[plain text format]

  

Fig 2: AUTOPHAGOSOME\_MATURATION(GO:0097352)
  
Blue-Pink O' Gram in the Space of the Analyzed GeneSet

  

Fig 3: AUTOPHAGOSOME\_MATURATION(GO:0097352): Random ES distribution
  
Gene set null distribution of ES for
 **AUTOPHAGOSOME\_MATURATION(GO:0097352)**
